# Optimized Reversed-Phase Liquid Chromatography/Mass Spectrometry Methods for Intact Protein Analysis and Peptide Mapping of Adeno-Associated Virus Proteins

**DOI:** 10.1089/hum.2021.046

**Published:** 2021-12-16

**Authors:** Ximo Zhang, Xiaoying Jin, Lin Liu, Zichuan Zhang, Stephan Koza, Ying Qing Yu, Weibin Chen

**Affiliations:** ^1^ Scientific Operations, Waters Corporation, Milford, Massachusetts, USA.; ^2^ Global CMC Development, Sanofi, Framingham, Massachusetts, USA.

**Keywords:** AAV, vector characterization, AAV peptide mapping, AAV protein analysis, AAV attribute monitoring, AAV analytical development

## Abstract

Recombinant adeno-associated viruses (AAVs) have emerged as the leading gene delivery platform owing to their nonpathogenic nature and long-term gene expression capability. The AAV capsid, in addition to protecting the viral genome, plays an important role in viral infectivity and gene transduction, indicating the value of the constituent viral proteins (VPs) being well-characterized as part of gene therapy development. However, the limited sample availability and sequence homology shared by the VPs pose challenges to adapt existing analytical methods developed for conventional biologics. In this study, we report the development of reversed-phase liquid chromatography/mass spectrometry-based methods for characterization of AAV capsid proteins at intact protein and peptide level with reduced sample consumptions. The developed methods allowed the measurement of VP expression with fluorescence detection and intact mass/post-translational modifications (PTMs) analysis through a benchtop time-of-flight mass spectrometer. The general applicability and validity of the methods for gene therapy product development were demonstrated by applying the optimized methods to multiple common AAV serotypes. A 1-h enzymatic digestion method was also developed using 1.25 μg of AAV VPs, providing >98% protein sequence coverage and reproducible relative quantification of various PTMs of the VPs. The efficient and sensitive analyses of AAV capsid proteins enabled by the reported methods provide further understanding and offer guidance in the development and manufacturing of AAV-related therapeutics.

## INTRODUCTION

Gene therapy refers to the modification or manipulation of gene expression or the genetic alteration of living cells for therapeutic purposes.^1,2^ Viral vectors, a linchpin of many gene therapies, have the primary functions of protecting the encapsulated genetic payload (RNA or DNA) and engaging in cellular targeting and trafficking.^1,3^ The most efficient viral vectors emerging from preclinical and clinical studies are adeno-associated virus (AAV), lentivirus, and adenovirus, among which AAV is the most explored owing to its lower risk in humans and efficient transduction in a variety of cells and tissues.^1,3,4^

AAV is a nonenveloped, single-stranded DNA parvovirus with many wild types found in nature. Structurally, AAV is an ∼26-nm diameter icosahedral capsid assembled from 60 viral protein (VP) monomers arranging into pentameric substructures.^5^ Each capsid contains three highly homologous VPs (VP1, VP2, and VP3) in a 1:1:10 proportion, where VP2 (∼65 kDa) comprises the entire amino acid sequence of VP3 (∼60 kDa) with an N-terminal extension, and VP1 (∼80 kDa) is an N-terminal extension of VP2.^5,6^ To date, at least eight distinct serotypes of AAV have been used for gene therapy.^1^ Although those AAV serotypes generally display 51–99% sequence homology, the differences in primary sequence of their VPs confer unique binding affinity toward various host cell receptors, leading to diverse tissue tropism.^5^

The continuous advancement and the expanding product pipelines of AAV-based gene therapeutics present challenges to product characterization.^7^ Similar to conventional biologics, well-characterized AAVs are required to meet predetermined specifications and regulatory standards for purity, potency, and safety.^7^ This industry demand calls for analytical technologies that are precise and accurate to monitor product quality and ensure batch-to-batch consistency. In addition, more AAV therapeutics progressing from early discovery to clinical development, robustness, validity, and ease-of-use of the analytical methods become increasingly important to ensure the smooth transit into late-stage development and commercialization. One of the challenges in the analytical testing of recombinant (r)AAV vectors is the high degree of structural complexity. The multimeric nature and the variations of individual VPs make the structure of AAVs more complex than many monomeric recombinant protein therapeutics. To add further complication, the whole rAAV particle consists of not only proteins but also genetic materials. This clearly entails the development of methods beyond those applied to more established modalities to ensure that the unique nature of AAV biology is fully addressed.

To characterize the rAAV vectors, X-ray crystallography and cryo-electron microscopy (cryo-EM) have been used to determine the three-dimensional (3D) structures of multiple AAV serotypes, showing only the VP3 common sequence is ordered.^5^ Although unveiling critical structural information of the virions, these studies on 3D structures only provide a “snapshot” of the capsid topology in a low-energy state. Efforts have been put forth to perform molecular level studies that are typically undertaken throughout the development of conventional biotherapeutics.^8–11^ One of the focuses is to establish in-depth understanding of capsid composition (*e.g.,* post-translational modifications [PTMs]) and their potential impact on viral infectivity and efficacy. This endeavor typically uses physicochemical methods such as mass spectrometry (MS) or liquid chromatography (LC) as they do not require product-specific analytical reagents such as monoclonal antibodies.^12^ Rumachik *et al.* applied multiple analytical approaches including MS, isoelectric focusing, and cryo-EM, to characterize AAV8 samples produced by two manufacturing platforms, human HEK293 cells and *Spodoptera frugiperda* (Sf9) insect cells.^8^ These rAAV8 samples differed in PTMs, including glycosylation, acetylation, phosphorylation, and methylation, which shed lights on their observed potency differences in various target-cell types. Giles *et al.* applied tandem mass spectrometry (MS/MS) to investigate asparagine and glutamine deamidation on AAV8 and demonstrated the potential impact of deamidation on transduction activity.^9^ More importantly, the authors explored the mutational strategies to stabilize the amine groups and improve vector performance. To support AAV-based gene therapy development, Jin *et al.* developed a well-executed approach for intact VP and peptides characterization using reversed-phase (RP) LC-MS, enabling the direct mass measurement and peptide sequence confirmation of 13 AAV serotypes for identification and purity assessment.^10^ In addition, a hydrophilic interaction liquid chromatography (HILIC)-MS–based method for intact VP analysis has been reported by Liu *et al.*^11^ Using a silica-hybrid–based amide HILIC column^13^ and trifluoroacetic acid (TFA) as a mobile phase modifier, the separation was performed on a wide range of AAV serotypes, and the MS sensitivity was enhanced by modifying the MS desolvation gas with propionic acid and isopropanol. Although all these methods are somewhat different, they all render insights into AAV sample quality and highlight the power of LC/MS as an effective analytical tool to facilitate the development of gene therapy products.

Besides the structural complexity, multiple challenges are still to be addressed for the analyses of rAAVs compared with typical recombinant proteins. The difficulties in large-scale manufacturing and purification of AAVs result in low yield of samples.^7^ This can be very problematic in supporting process development where often only micrograms of rAAVs are available for the analyst to cover a range of required assays. Another challenge is the low concentration of rAAV samples. As examples, Luxterna^®^, an ocular therapy, is formulated at 5 × 10^12^ vector genomes per milliliter (vg/mL). This translates into protein concentrations of 30 μg/mL if 100% capsids contain transgene. With only 8% relative abundance, VP1 and VP2 are at low microgram levels in the formulated samples, increasing the risk of protein loss during sample preparation and analysis. As such, a sensitive and robust method that meets the challenge of structure complexity and sample scarcity of rAAV while delivering insightful information on product quality attributes is highly desirable.

In this study, we report our work in the characterization of rAAV capsid using LC-MS techniques with an aim to develop robust, versatile, and sample-sparing methods that require minimal expertise to support the ever-growing activities in rAAV process development and manufacturing. We extended these analyses to encompass rAAV serotypes that show clinical promises and found broad applicability of the methods in measuring the critical quality attributes such as VP stoichiometry and the extent of PTMs (*e.g.,* deamidation and oxidation) of capsid proteins.

## EXPERIMENTAL SECTION

### Intact protein analysis of AAV vectors by LC-fluorescence/MS

Multiple serotypes of rAAV vectors were obtained from Vigene Bioscience (Rockville, MD), including AAV1, AAV2, AAV5, AAV6, AAV8, and AAV9. Additional AAV5 samples were produced in Sanofi. AAV vectors were diluted with Milli-Q water to a final titer of 1 × 10^13^ vg/mL or used as is if the received sample concentration was lower. As previously reported,^10^ AAV vectors were treated with acetic acid (Sigma-Aldrich, St. Louis, MO) at 10% (v/v) concentration for 15 min, then centrifuged at 12,000 rpm for 5 min to ensure complete capsid dissociation.

The LC-fluorescence (FLR)/MS analysis of AAV capsid proteins was performed on a BioAccord LC-MS system (Waters Corp., Milford, MA), which consists of an Acquity UPLC I-Class PLUS system, an Acquity FLR detector, and an Acquity RDa time-of-flight (ToF) mass detector. Ten microliters (∼0.5 μg of protein) of AAVs was injected onto an Acquity BEH C4 column (2.1 × 100 mm, 1.7 μm, 300 Å; Waters Corp.) maintained at 80°C. The mobile phases were 0.1% LC-MS grade difluoroacetic acid (DFA; Waters Corp.) in water (A) and acetonitrile (B). At a flow rate of 0.2 mL/min, the gradient was set to start from 20% B and ramped to 32% B over 1 min, 32–36% B in 17 min, 36–80% B in 20 min and maintained at 80% B until 21.5 min, and 20% B from 22 to 30 min for re-equilibration.

Mass detection was carried out on the RDa mass detector using electrospray ionization (ESI) in positive ion mode. The MS settings were as follows: capillary voltage, 1.5 kV; cone voltage, 65 V; desolvation temperature, 550°C; scan range, 400–7,000 *m/z*; and scan rate, 2 Hz. MaxEnt1 algorithm within the waters_connect informatics platform was used to process the raw MS data of intact VPs to yield the deconvoluted masses. For VP stoichiometry measurement, 1 μL (∼0.05 μg of proteins) of AAVs was injected under FLR detection with 280 nm as the excitation wavelength and 350 nm as the emission wavelength at 2 Hz scan rate.

### Enzymatic digestion of AAV5 VPs

Before enzymatic digestion, denaturing size-exclusion chromatography (SEC) was used to remove the surfactant, Tween 20, from the AAV samples. Twenty-five microliters of AAV5 sample (1 × 10^13^ vg/mL) were injected onto a Acquity BEH SEC200 column (2.1 × 150 mm, 1.7 μm, 200 Å; Waters Corp.) maintained at 23°C. A mobile phase containing 0.1% TFA, 0.1% formic acid (FA), 10% acetonitrile, 20% isopropanol alcohol, and 69.8% water (all solvents and additives were from Fisher Scientific, Waltham, MA) was used at a flow rate of 0.08 mL/min. The eluted AAV5 VPs was manually collected postcolumn from 2 to 4 min, to which 5 μL of 1 mM methionine solution was added and mixed in a 0.5-mL Eppendorf Protein Lobind tube (Hamburg, Germany). The mixture was immediately placed in a −80°C freezer for rapid freezing, and then lyophilized using a CentriVap vacuum concentrator (Labconco, Kansas City, MO) within 1 h. The dried AAV5 VPs were reconstituted in 5 μL of buffer solution consisting of 0.05% (w/v) *Rapi*Gest denaturant (Waters Corp.), 0.5 mM dithiothreitol (DTT; Fisher Scientific), 0.1 mM ethylenediaminetetraacetic acid (EDTA; Sigma-Aldrich), and 50 mM pH8.0 Tris-HCl buffer (Fisher Scientific). The reconstituted AAV5 VPs was incubated at 70°C for 3 min for denaturation. After cooling down to room temperature, the denatured AAV5 VPs solution was mixed with 2 μL of 0.1 μg/μL sequence grade-modified trypsin (Promega, Madison, WI) and kept at 37°C for 1 h for proteolytic digestion. The digested AAV5 sample was then diluted using 18 μL of 10 mM methionine (in water) solution and placed in sample manager at 4°C for LC-MS analysis.

### Ultrahigh-performance liquid chromatography-MS peptide mapping

The LC-MS analysis of AAV peptides was performed on the BioAccord System with the same configuration as specified in the section of intact mass analysis. Twenty microliters (∼1 μg of proteins) of the tryptic digest of AAV VPs were injected onto an Acquity BEH C18 column (2.1 × 100 mm, 1.7 μm, 300 Å; Waters Corp.) maintained at 65°C. The peptides were separated using a mobile phase containing 0.1% LC-MS grade FA (Fisher Scientific) in water (A) and acetonitrile (B). At a flow rate of 0.2 mL/min, the gradient was set as 1% B for 3 min, then ramped from 1% to 15% B in 18 min, 15 to 30% B in 48 min, 30 to 55% B in 51 min, 55 to 95% B in 65 min and maintained at 95% B until 67 min, and 1% B from 70 to 85 min for equilibration.

MS data were collected on the RDa detector under the “Full scan with fragmentation” mode. In this acquisition mode, both low-energy peptide precursor and the corresponding high-energy fragmentation data are acquired simultaneously. The other MS settings were as follows: capillary voltage, 1.2 kV; cone voltage, 20 V; fragmentation cone voltage, 60–120 V; desolvation temperature, 350°C; scan range, 50–2,000 *m/z*; and scan rate, 2 Hz. A SYNAPT-XS Quadrupole ToF mass spectrometer (Waters Corp.) was also used for sequence confirmation and PTM identification with the following settings: capillary voltage, 2.2 kV; source temperature, 120°C; collision energy, 20–50 eV; desolvation temperature, 350°C; desolvation gas flow, 500 L/h; scan range, 50–2,000 *m/z*; and scan rate, 2 Hz. Targeted MS/MS was used for the sequence confirmation of low-abundance N-terminal peptides with the collision energy ramping at 30–50 eV. MS data were processed using the peptide mapping workflow within the waters_connect informatics platform. Mass tolerance was set as 10 ppm for precursor ions and 20 ppm for fragmentation ions. Up to one miscleavage with a minimum of three b-/y-ions matches were set as the criteria for peptide identification. PTMs including *N*-acetylation, deamidation (Asn and Gln), methylation, phosphorylation, and oxidation (Met and Trp) were considered as potential amino acid modifiers. The combined MS response of the isotopic peaks from detected charge states was used for PTM quantification with the assumption of all isotopes having the same ionization efficiency.

## RESULTS AND DISCUSSION

### Method development for intact mass analysis of AAV8 VPs by RPLC-MS

**Figure 1.**
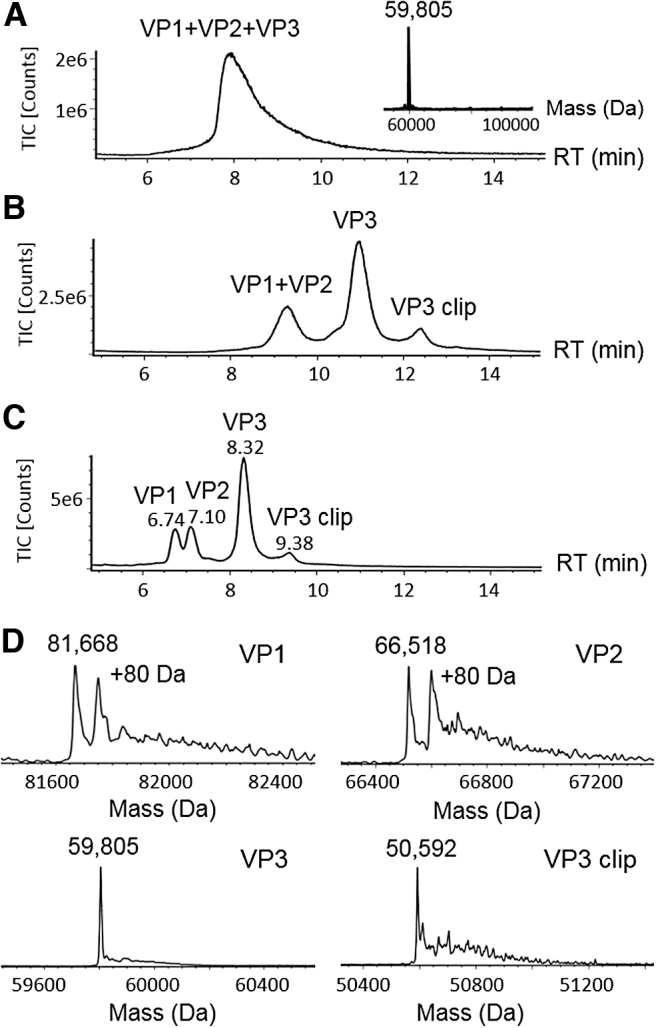
RPLC-MS method development of AAV8 capsid proteins. **(A)** Separation of VPs using formic acid as mobile phase modifier on a BEH C8 column; **(B)** separation using DFA as mobile phase modifier on the C8 column and **(C)** a BEH C4 column; **(D)** deconvoluted MS spectra of VP1, VP2, VP3, and the VP3 variant (labeled as “VP3 clip”). An isoform with plus 80 Da mass shift was detected in the deconvoluted spectra of VP1 and VP2 and identified as phosphorylation. AAV, adeno-associated virus; DFA, difluoroacetic acid; MS, mass spectrometry; RPLC, reversed-phase liquid chromatography; VP, viral protein.

The LC/MS analysis of AAV capsid proteins is complicated by their disparate abundances, sequence homology, and the limited sample availability. Conventional RPLC-MS methods are in favor of FA as the mobile phase additive for better MS responses. However, RP separation with FA cannot chromatographically resolve the AAV VPs,^10^ requiring micrograms of AAV materials to obtain acceptable MS signal on the low-abundance species. As given in Fig. 1A, when analyzing AAV8 capsid proteins using mobile phases containing FA on a C8 column, all VPs co-eluted as a single peak in the total ion chromatogram (TIC). The masses of VP1 (∼80 kDa) and VP2 (∼65 kDa) were not observed in the deconvoluted MS spectra (Fig. 1A, inset), which may be attributed to ion suppression caused by the co-eluting abundant VP3 peak (∼60 kDa) and the low ion intensity caused by the poor chromatographic peak shape. As a widely used ion-pairing reagent in RP protein/peptide separation, TFA improves protein separation by mitigating secondary interactions but can cause significant MS ion suppression, thus requiring higher mass loading in LC/MS analysis.

To improve the LC/MS analysis of AAV VPs, an RPLC-MS method was developed using an alternative ion-pairing reagent, DFA,^14^ as the mobile phase additive. As a partially fluorinated carboxylic acid, DFA offers similar ion-pairing effect but is more volatile compared with TFA, thus providing a balanced outcome in chromatographic separation and MS sensitivity. As given in Fig. 1B, the previously co-eluting VP1 and VP2 were resolved from VP3. In addition, an extra peak was observed at 12.2 min, which was assigned as a VP3 fragment (labeled as “VP3 clip”) based on its deconvoluted mass upon MaxEn1 data processing. In addition to the resolution improvement in VP separation, the ion intensity of VP3 was increased owing to the narrower chromatographic peaks compared with the separation using FA.

To further optimize the separation, we evaluated the columns with different bonded phases, such as C4 and C18, and different pore sizes on the particle substrates. On a C4 column, VP1 and VP2 were further separated (Fig. 1C) partially because of the increased selectivity afforded by the less hydrophobic C4 ligand. In addition to surface chemistry, pore size of the packed particles played a key role in the improvement of separation. Compared with the C8 column packed with 130 Å pore size particles (Fig. 1B), the wide pore (300 Å) C4 column further reduced the peak widths (Fig. 1C), which may in part be attributed to the increased surface interactions and mass transfer offered by the larger pores.

The improved chromatographic separation facilitates the MS analysis of individual AAV VPs on a benchtop MS system. Figure 1C shows an LC/MS chromatogram with 0.5 μg of AAV8 sample loaded on column. The masses for peaks at 6.74, 7.10, and 8.32 min were calculated to be 81,668, 66,518, and 59,805 Da upon MS charge deconvolution (Fig. 1D). The observed masses were agreeable with the theoretical masses of VP1, VP2, and VP3 (Supplementary Table S1),^10^ suggesting that the developed method is capable of measuring intact VP masses. The accurate mass measurement provides a means to deduce the potential PTMs on the VPs including acetylation and phosphorylation. For example, VP1 and VP3 each show a mass difference of 89 Da between the measured mass and the corresponding theoretical mass, suggesting the truncation of N-terminal methionine and the addition of N-terminal acetylation as the potential molecular form,^10^ whereas the peaks showing 80 Da mass shift on VP1 and VP2 were attributed to their phosphorylated proteoforms. The peak eluting at 9.38 min was found to have a mass of 50,592 Da, matching the mass of a VP3 fragment resulting from the hydrolysis at the acid-labile Asp_659_-Pro_660_ bond.^15^ In comparison with other separation modes such as HILIC^11^ for intact VP analysis, the developed RPLC-MS method has the flexibility to readily adjust the injection volumes, where low-concentration AAV samples can be directly injected without the need to preconcentrate or change the sample matrix. The downside of RPLC is that the phosphorylated VPs co-elute with their native forms and must be quantified by MS responses.

### Determination of VP ratios

**Figure 2.**
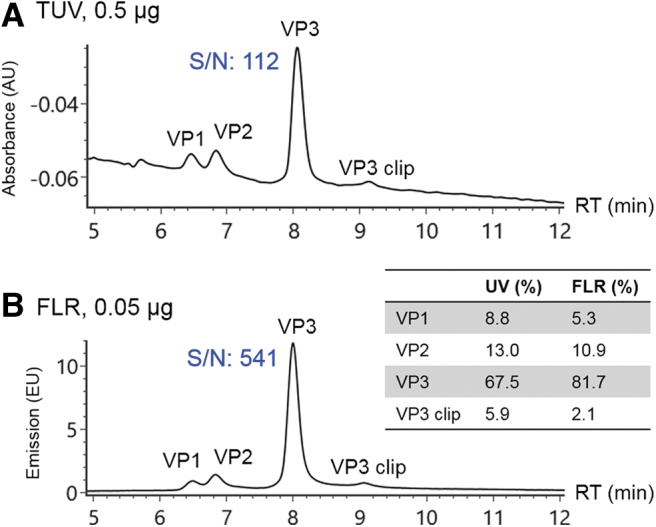
Stoichiometry of AAV8 VPs was measured under optical detection including **(A)** UV (wavelength at 280 nm), and **(B)** FLR (excitation wavelength at 280 nm, emission wavelength at 50 nm) with annotated peak assignment and S/N. *Inset* table showed calculated relative abundances of the VPs. With a 10-fold lower mass load, the S/N of VP3 under FLR detection was five times higher than UV detection, suggesting ∼50-fold enhancement in sensitivity. FLR, fluorescence; UV, ultraviolet.

The manufacturing process of AAV vectors can impact the VP stoichiometry, which may result in different potencies.^16–18^ Thus it is necessary to monitor the capsid protein ratios during the development of AAV-based therapeutics to ensure batch-to-batch consistency. Typically, optical detection signals such as ultraviolet (UV) or FLR are preferred over MS in protein quantification for higher robustness and more extended linear dynamic range. The improved resolution by the developed RPLC method allows calculation of relative abundance among individual VPs based on optical detection signals. Figure 2A shows the resolved VPs using UV detection. The VP ratios were calculated to be 9:13:74 for VP1:VP2:VP3 based on integrated peak area (Fig. 2, inset). The sensitivity of measurement was enhanced by monitoring protein intrinsic FLR (Fig. 2B).^19^ As given in Fig. 2B, the S/N of VP3 is 512 at 0.05 μg mass load with FLR detection, whereas an S/N of 112 for VP3 at 0.5 μg is acquired under UV detection, demonstrating an ∼50-fold improvement in sensitivity. As expected, the protein ratios measured by FLR signals (5:11:84 for VP1:VP2:VP3) were different from UV detection because of the changes in detection mechanism (Fig. 2, inset). Specifically, the difference of protein ratios in FLR and UV measurement can be attributed to the number and response factor of the aromatic amino acid residues (Trp, Tyr, and Phe) contained in the VP molecules,^20^ as well as the presence of phosphate groups in VP1 and VP2, which can quench the FLR of tryptophan.^21^ Thus, lower ratios of VP1 and VP2 were observed in FLR signals.

### Analyzing additional AAV serotypes

**Figure 3.**
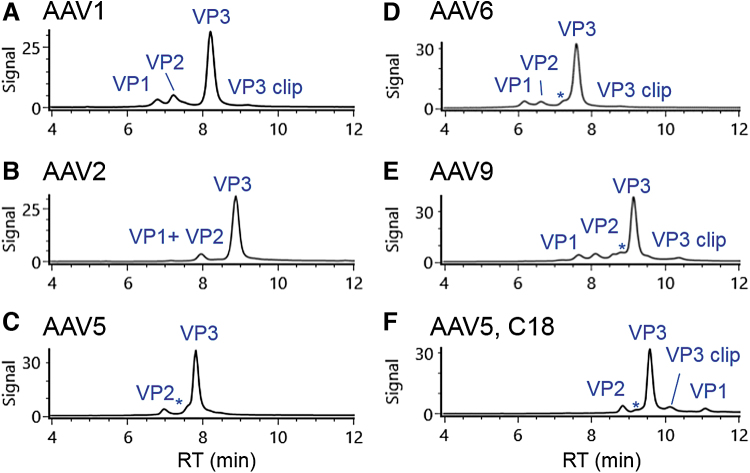
Separation of additional five AAV serotypes including **(A)** AAV1, **(B)** AAV2, **(C)** AAV5, **(D)** AAV6, **(E)** AAV9, and **(F)** AAV5 separated using C18 column. Peaks annotated with * are the observed variants with the same mass of VP3 in each AAV sample.

The method initially developed for AAV8 was applied to five additional serotypes of AAV (1, 2, 5, 6, and 9) to examine its general applicability in gene therapy product development. Figure 3 shows that the majority of these AAV serotypes (AAV1, AAV6, and AAV9) have a comparable chromatographic profile as that for AAV8, although the retention times of VPs vary by serotype. A front shoulder peak (annotated with *) of VP3 was also observed on most AAV serotypes analyzed in the study. The deconvoluted mass for the shoulder peak is the same as that of the main VP3 peak, suggesting it is likely a VP3 isomer. Using the described chromatographic conditions, the VP1 and VP2 from AAV2 co-eluted in the analysis (Fig. 3B), implying a shallower gradient or alternative separation mode is needed to fully resolve those peaks. The separation of AAV5 VPs showed a similar profile as that of AAV2 except VP2 was observed in the MS spectra of the peak at 6.95 min (Fig. 3C), possibly because of the co-elution of VP1 with the abundant VP3 peak. When the AAV5 VPs were analyzed using a C18 column, the VP1 peak appeared after VP3 peak (Fig. 3F). This change in elution order between VP1 and VP3 for AAV5 is ascribed to the increased hydrophobicity of VP1 in AAV5, as was indicated by the presence of more hydrophobic residues (such as proline and phenylalanine) in its protein sequence. For all AAV serotypes, the observed masses of the VPs (Supplementary Table S2) were consistent with a previous report.^10^ In addition, phosphorylated and acetylated VP isoforms were also identified in the samples under analysis, demonstrating the general applicability of the optimized LC-FLR/MS methods for common AAV serotypes in gene therapy development.

**Figure 4.**
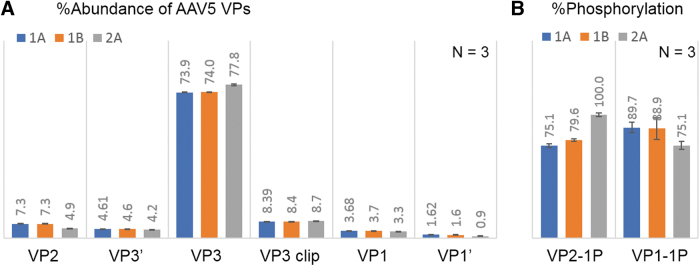
LC-FLR/MS analysis of AAV5 samples with potency differences, showing sample 2A (from the modified manufacturing process) is different from sample 1A and 1B (from original manufacturing process) regarding **(A)** %abundance of VPs, and **(B)** level of phosphorylation in VP2 and VP1. The phosphorylation level was normalized against the relative abundance of phosphorylated VP2 in sample 2A. VP3′ and VP1′ are detected variants of VP3 and VP1. The error bars represent the standard deviation of the relative abundances from three consecutive injections.

The change in manufacturing process can impact product quality attributes of AAV therapeutics, therefore comparability studies are undertaken to monitor the product consistency during process development and commercialization. To investigate the potency difference of AAV5 samples after a manufacturing change, a comparability study of three samples was conducted using the optimized RPLC-FLR/MS methods, including sample 1A and 1B from the original manufacturing process, and sample 2A from the modified process. Analyses were performed in triplicate to ensure the precision of measurement. In RPLC-FLR analysis (Supplementary Fig. S1), six peaks were resolved and aligned well across the three samples except the abundance of VP2 was lower in sample 2A. Figure 4A shows the ratio comparison of the VPs, confirming the consistency of sample 1A and 1B and the 2.3% lower VP2 along with increased VP3 in sample 2A. The results matched with the measurement by an orthogonal assay through capillary electrophoresis sodium dodecyl sulfate (CE-SDS) (Supplementary Fig. S2), implying the viability of RPLC-FLR as a routine method to monitor VP changes for potency assessment. In addition, identical masses were observed across samples for all VPs in LC-MS analysis (Supplementary Table S3), whereas a higher phosphorylation level of VP2 and a lower phosphorylation level of VP1 were observed in sample 2A (Fig. 4B). Although phosphorylation of AAV5 has not been reported to affect potency, the observed differences in phosphorylation level entail further investigation to evaluate the impact on other attributes of AAV5 such as transduction efficiency and intercellular trafficking.^8^ In summary, the RPLC-based methods provide efficient separation of intact VPs for AAV capsid analysis. The results demonstrated the reproducibility and flexibility of the method, making them readily adopted in various environments. The current method may require optimization to deliver more satisfying separation results for some AAV serotypes, and it faces challenges in separating oxidized isoforms from their native ones, which will need orthogonal detection methods such as MS, or other characterization methods such as peptide mapping to confirm the existence (or absence) of those species.

### Enzymatic digestion method development for AAV5 VPs

Enzymatic digestion of proteins followed by LC/MS analysis of the proteolytic digest is commonly used for sequence confirmation and PTM identification of protein therapeutics. Although a full sequence coverage of VPs has been demonstrated in previous report with multi-enzyme digestions,^10^ the work used 10–20 μg AAV VPs to prepare the protein digest. Such sample requirement for a single peptide mapping workflow is difficult to satisfy because of the limited availability of AAV sample during early development phase. However, enzymatic digestion with greatly reduced AAV materials faces multiple challenges in sample preparation. In addition to the restriction of low sample concentration, surfactants in AAV formulation buffers are problematic in MS analysis, such as poloxamer or tweens. Although these surfactants were separated from intact VPs and did not cause problem in previous RPLC-MS analysis, they can severely interfere with LC-MS analysis of peptides.

Typically, a buffer exchange step before digestion is needed to remove the surfactants along with enzyme inhibitors such as the denaturant and alkylation reagent.^22^ However, the complete removal of surfactants is challenging because their concentrations are usually above the critical micelle concentration. In addition, in common buffer exchange methods, such as spin filtering or dialysis, low protein concentration can lead to significant sample loss mostly because of the nonspecific adsorption to the membrane filter.^23^ Furthermore, in the case of low-concentrated AAV VPs (∼50 μg/mL), the attempt to use less material in analysis will result in two difficult scenarios where either a very small volume of AAV sample is taken, or a larger volume sample with low protein concentration. From a sample preparation perspective, the small sample volume means that many common buffer exchange devices/protocols cannot be readily adopted. On the contrary, low-concentrated protein samples would further decrease the enzymatic digestion rate and protein digest recovery. These challenges prevent the adoption of conventional enzymatic digestion protocols to the limited sample and call for a completely new sample preparation approach.

A new digestion method that is compatible with low microgram quantities of AAVs was developed for peptide mapping in this study. The protein loss was greatly reduced by minimizing buffer exchange and liquid transfer steps. Given the limited sample amount, the entire AAV capsid rather than the isolated VPs was selected to develop a single enzymatic digestion. To remove the surfactant from the AAVs, we developed an 8-min denaturing SEC-based method to separate the AAV VPs from the surfactant. Using AAV5 as an example, 1.25 μg of VPs were injected and collected within a 2 min window, resulting in a surfactant-free sample at a protein recovery over 95% (Supplementary Fig. S3). The fraction was lyophilized to dryness to remove organic solvents and increase the protein concentration for the following steps. Using 5 μL of reconstitution buffer that contains an MS-friendly denaturant, *Rapi*Gest,^24^ at 0.05% (w/v), a one-pot denaturation and digestion method was developed. Although the AAV5 VPs do not have disulfide bonds in theory,^25^ a reducing reagent, DTT, was included in the buffer at low level to avoid disulfide pairing. This buffer composition can improve the solubility of the denatured proteins with minimal impact on enzymatic activities, making the buffer exchange step unnecessary before digestion. In addition, with the presence of the denaturant and reducing agent in the digestion buffer, alkylation was not required to prevent the reformation of disulfide bonds, which in turn eliminated the need for an additional buffer exchange.

The digestion method was further optimized for trypsin-based proteolysis of AAV5 to minimize the sample preparation artifacts and digestion miscleavages. To reduce artifactual oxidation, 10 mM methionine was added as an oxygen scavenger to the collected AAV VPs from denatured SEC fractionation before lyophilization. To further improve the PTM quantification, DTT was selected over tris(2-carboxyethyl)phosphine (TCEP) as the reducing agent as TCEP can react with the oxidized methionine residues that occur before digestion.^26^ It was reported that the presence of DTT in the buffer can cause methionine oxidation because of the formation of hydrogen peroxide from metal-catalyzed reduction.^27^ Therefore, we added EDTA as a metal chelator and decreased the concentration of DTT in digestion solution to 0.5 mM. This DTT concentration is ∼10-fold less than the concentration commonly used in the digestion methods for monoclonal antibodies.^28^ Despite the lower concentration used in the current method, the molar ratio of DTT to the cysteine residues in AAV5 was still excessive (>25:1 ratio) to prevent the potential formation of disulfide bonds. The other digestion conditions were optimized to achieve a balance between peptide miscleavages and method-induced modifications. To facilitate a more complete digestion, denaturation of AAV5 VPs was carried out at 70°C using a surfactant, *Rapi*Gest, as the denaturant to unfold and stabilize the proteins^24^ before the tryptic digestion that was conducted at 37°C with 1:10 enzyme-to-substrate ratio. The presence of the surfactant in the digestion mixture leads to more exposed sites for enzymatic cleavages, which makes it possible to reduce the reaction time from typical overnight digestions.^24^ To minimize sample preparation artifacts, for example, oxidation and deamidation,^22^ the denaturation and digestion times were optimized to be 3 min and 1 h, respectively, resulting in a total sample preparation time of 2.5 h. This optimized digestion method grants relatively flexible protein consumption. However, considering the low abundance of VP1 and VP2 on AAV capsid (<10%), the described 1.25 μg protein digest has generally precluded the use of UV detection, and is approaching the detection capability of the ultrahigh-performance liquid chromatography (UHPLC)/MS instrument configuration used for this study. Although peptide mapping at analytical scale is preferred for robustness purposes, the sample consumption in the digestion might be further reduced to meet the need of other analyses such as nano LC/MS.

### UHPLC/MS analysis of AAV5 VPs tryptic digests

**Figure 5.**
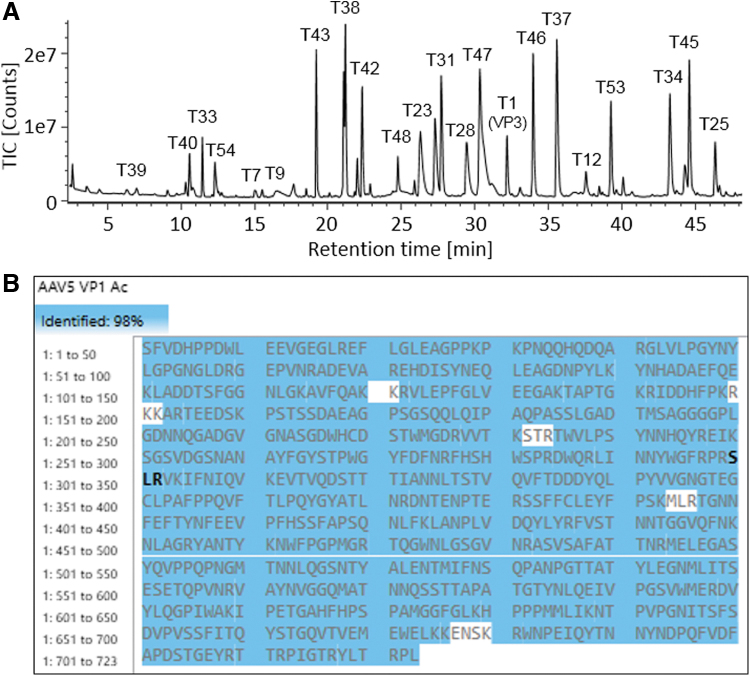
Peptide analysis of AAV5 VPs using ∼1.25 μg proteins as the starting material in enzymatic digestion. **(A)** TIC of AAV5 tryptic digest with major peaks annotated with corresponding peptide names. **(B)** Peptide map showed 98% sequence coverage of AAV5 VP1 with identified peptides highlighted in *blue*. Data were processed in UNIFI peptide mapping workflow. TIC, total ion chromatogram.

**Figure 6.**
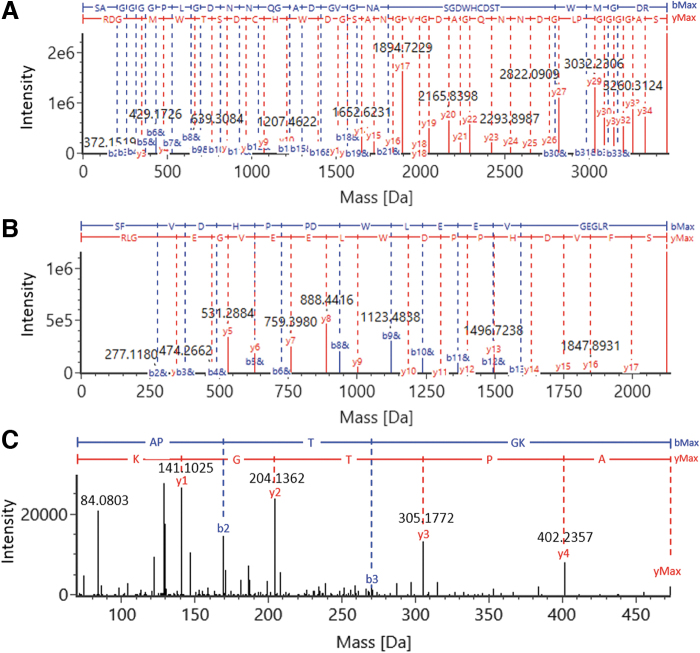
Identification of N-terminal peptides of AAV5 VPs via MS/MS. The identified primary ions were shown in the MS fragmentation spectra of **(A)** VP3 N-terminus (Ac)SAGGGGPLGDNNQGADGVGNASGDWHCDSTWMGDR, **(B)** VP1 N-terminus (Ac)SFVDHPPDWLEEVGEGLR, and **(C)** VP2 N-terminus APTGK. The fragmentation spectra of the singly charged VP2 N-terminus was obtained by target MS/MS at a higher collision energy. Annotation in *blue* and *red* indicate the observed b-ions and y-ions, respectively. MS/MS, tandem mass spectrometry.

The digested AAV5 VPs were analyzed on the benchtop UHPLC-MS system to validate the developed digestion protocol for AAV protein sequence coverage. Using a 45-min gradient, the peptides were well separated on a C18 RP column with intensive MS signals shown in the TIC trace (Fig. 5A). The peptide identities were assigned based on observed masses and high-energy fragmentation ions. The coverage map of AAV5 VP1 shows a 98% coverage of the protein sequence with the identified peptides highlighted in blue (Fig. 5B). The sequence coverages of VP2 and VP3 were both at 98% as well, as the only distinct peptide between their polypeptide backbones were those derived from their N-termini. The deconvoluted MS fragmentation spectrum of VP3 N-terminus is given in Fig. 6A with the annotated precursor mass (3460.3516 Da) and extensively distributed b-/y-ion series, confirming the peptide sequence of SAGGGGPLGDNNQGADGVGNASGDWHCDSTWMGDR. The MS data also showed a +42 Da mass shift compared with the theoretical mass of VP3 N-terminal peptide (mass accuracy of 0.2 ppm), suggesting the existence of *N*-acetylation associated with the peptide. The y_34_ and y_max_ ions with the 42 Da mass addition on the serine residue confirms acetylation taking place at the N-terminus. Similarly, the VP1 N-terminal peptide, SFVDHPPDWLEEVGEGLR (Fig. 6B) was identified with the mass accuracy of 2.2 ppm for the precursor, and *N*-acetylation was also found occurring on the serine residue. The VP2 N-terminal peptide, APTGK, consisting of only five amino acid residues, is only weakly retained on the RP column. In addition, this singly charged VP2 N-terminal peptide does not readily fragment under the general MS fragmentation settings used in the data independent acquisition mode. Hence, target MS/MS data acquisition mode and a higher collision energy were used to generate more extensive b- and y-ion fragments to confirm the peptide identity (Fig. 6C).

**Figure 7.**
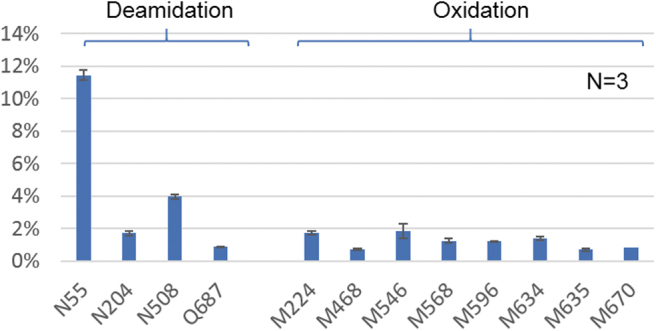
Identified PTMs with >0.5% relative abundance including deamidation on N55, N204, N508, Q687; and oxidation on M224, M468, M546, M508, M596, M635, and M670. The error bars represent the standard deviation (<0.5%) of %PTMs from three separate sample preparations. PTM, post-translational modification.

It was reported that the PTMs on AAV capsid proteins can impact the transduction efficiency and tropism of the virus,^8,9,29^ therefore the determination of PTMs are often a focus of peptide analysis. Using the developed method, a series of PTMs were identified on all three VPs, including *N*-acetylation, deamidation, oxidation, methylation, and phosphorylation (Supplementary Fig. S4), providing additional confirmation of the observed phosphorylation in intact mass analysis. These PTMs were quantified based on the MS responses of the modified and native peptides, where the sites with >0.5% deamidation and oxidation are given in Fig. 7. Deamidation on most asparagine residues was found <5% with the exception that N55 residue on VP1, which was reported to potentially impair transduction efficiency,^4,9^ showed >10% deamidation. Oxidation is a common degradation pathway of proteins and was found to be <2% on all methionine residues as expected.^8^ Although the clinical impact of AAV methylation has not been reported, multiple methylated lysine and arginine residues were observed on AAV5. Not surprisingly, miscleavage was often noticed to occur on the peptides bearing methylation, which might be because of the steric hinderance of the methyl group on lysine or arginine residues. Because the miscleaved peptides can have different MS ionization efficiencies, the level of methylation estimated in this study can be more accurately determined through alternative enzymatic digestions such as Asp-N in future work. To evaluate the method reproducibility, digestions were performed in triplicate and <0.4% standard deviation was observed for all PTMs with the level >0.5% (Fig. 7). With reproducible sequence confirmation and quantification of PTMs, the optimized digestion method provides a means to analyze microgram amounts of AAV materials at the peptide level for identification, characterization, and product consistency purposes.

In conclusion, multiple LC and LC/MS-based methods were developed and optimized to deliver more comprehensive characterization of AAV capsid proteins. The efficient separation afforded by the intact RPLC methods using DFA as the mobile phase additive allowed the VP stoichiometry consistency to be measured using 50 ng of AAV and FLR detection, and intact mass and PTM analyses of 0.5 μg of AAV when using a benchtop ESI-ToF-MS detector. Analysis of several AAV serotypes demonstrated the general applicability of the method for routine use such as comparability studies. Using a combination of denaturing SEC fractionation and MS-friendly detergent, *Rapi*Gest, the optimized 1-h enzymatic digestion method can generate high quality of tryptic digests with only 1.25 μg of AAV sample where nearly 100% sequence coverage is achieved for all VPs and several critical PTMs were identified and reproducibly quantified through a benchtop ToF-MS system. Therefore, the efficiency and sensitivity provided by these methods can benefit the analyses of AAV capsid proteins to improve the understanding and guide the design and manufacturing of new AAV therapeutics.

## Supplementary Material

Supplemental data

Supplemental data

Supplemental data

Supplemental data

Supplemental data

Supplemental data

Supplemental data
