# Supplementary material for: Optimized Reversed-Phase Liquid Chromatography/Mass Spectrometry Methods for Intact Protein Analysis and Peptide Mapping of Adeno-Associated Virus Proteins
Source: Hum Gene Ther. 2021 Dec 16;32(23-24):1501–11. doi: 10.1089/hum.2021.046 (PMC8742267; doi:10.1089/hum.2021.046)
Supplement: Supplemental data [file Suppl_TableS1.pdf]

Peak	**Tentative identity**	**AA sequence**	**Observed mass (Da)**	**Theoretical mass (Da)**
1	VP1	2(Ac)-738	81,668	81,667
2	VP2	139-738	66,518	66,519
3	VP3	205(Ac)-738	59,805	59,805
4	VP3 clip	205(Ac)-659	50,592	50,593

Table S1. Observed (average) mass, theoretical amino acid sequence, and theoretical masses of the separated VPs in Figure 1 using the RPLC-MS methods.
