# Supplementary material for: Optimized Reversed-Phase Liquid Chromatography/Mass Spectrometry Methods for Intact Protein Analysis and Peptide Mapping of Adeno-Associated Virus Proteins
Source: Hum Gene Ther. 2021 Dec 16;32(23-24):1501–11. doi: 10.1089/hum.2021.046 (PMC8742267; doi:10.1089/hum.2021.046)
Supplement: Supplemental data [file Suppl_TableS2.pdf]

Serotype	VP1			VP2			VP3		
	Observed mass (Da)	AA sequence	Theoretical mass (Da)	Observed mass (Da)	AA sequence	Theoretical mass (Da)	Observed mass (Da)	AA sequence	Theoretical mass (Da)
**AAV1**	81,289	2(Ac)-736	81,286	66,096	139-736	66,093	59,517	204(Ac)-736	59,517
**AAV2**	81,854	2(Ac)-735	81,856	66,486	139-735	66,488	59,974	204(Ac)-735	59,974
**AAV5**	80,336	2(Ac)-724	80,336	65,283	139-724	65,283	59,463	199(Ac)-724	59,463
**AAV6**	81,324	2(Ac)-736	81,322	66,094	139-736	66,096	59,518	204(Ac)-736	59,519
**AAV8**	81,668	2(Ac)-738	81,667	66,519	139-738	66,519	59,805	205(Ac)-738	59,805
**AAV9**	81,292	2(Ac)-736	81,291	66,210	139-736	66,210	59,732	204(Ac)-736	59,733

Table S2. Observed (average) mass, theoretical amino acid sequence, and theoretical masses of the capsid proteins from six AAV serotypes analyzed using the RPLC-MS methods.
