# Supplementary material for: Optimized Reversed-Phase Liquid Chromatography/Mass Spectrometry Methods for Intact Protein Analysis and Peptide Mapping of Adeno-Associated Virus Proteins
Source: Hum Gene Ther. 2021 Dec 16;32(23-24):1501–11. doi: 10.1089/hum.2021.046 (PMC8742267; doi:10.1089/hum.2021.046)
Supplement: Supplemental data [file Suppl_FigureS1.pdf]

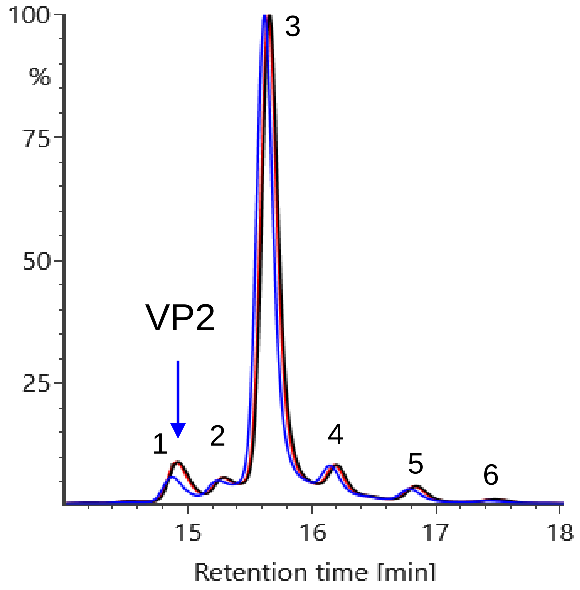

Figure S1. Overlaid chromatograms of sample 1A (black trace), 2B (red trace), and 2A (blue trace). Peaks were annotated based on the observed mass in Table S2. Additional two peaks were observed on all three samples and annotated as VP3’ and VP1’, which might be the structural isomer of VP3 and phosphorylated VP1, respectively.
