# Supplementary material for: Optimized Reversed-Phase Liquid Chromatography/Mass Spectrometry Methods for Intact Protein Analysis and Peptide Mapping of Adeno-Associated Virus Proteins
Source: Hum Gene Ther. 2021 Dec 16;32(23-24):1501–11. doi: 10.1089/hum.2021.046 (PMC8742267; doi:10.1089/hum.2021.046)
Supplement: Supplemental data [file Suppl_FigureS2.pdf]

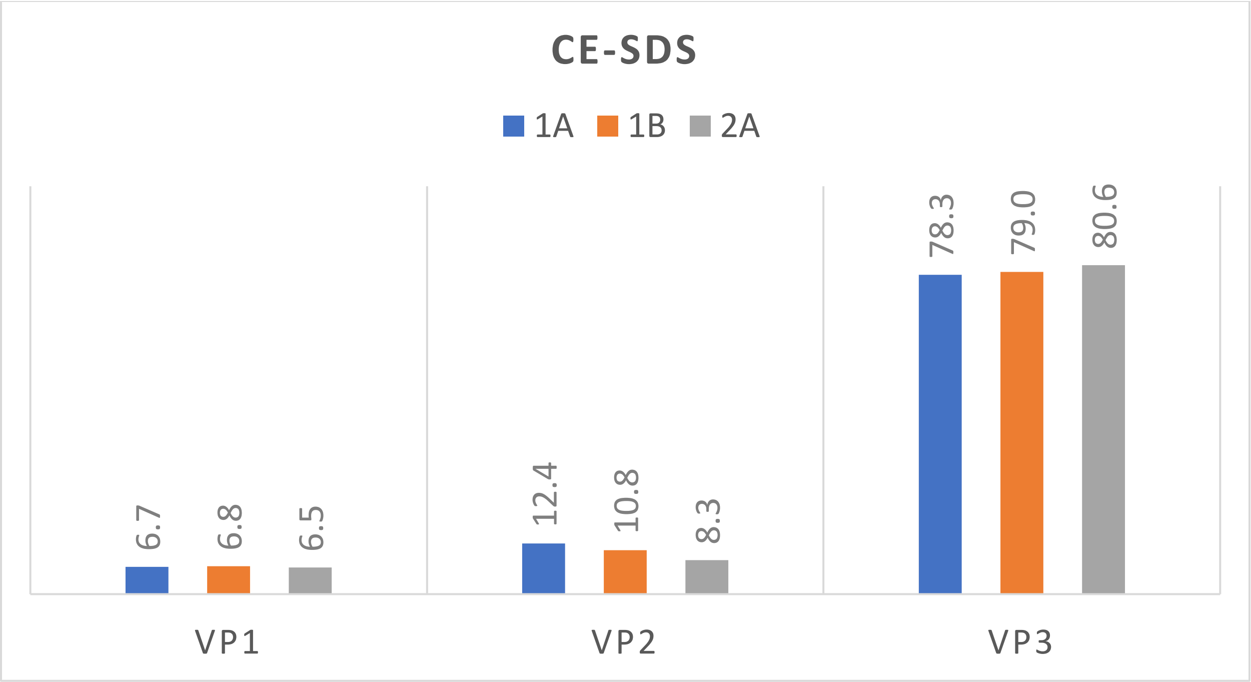

Figure S2. CE-SDS analysis of AAV5 samples with potency differences, showing sample 2A (from the modified manufacturing process) is different from sample 1A and 1B (from original manufacturing process) regarding %abundance of VPs.
