# Supplementary material for: Optimized Reversed-Phase Liquid Chromatography/Mass Spectrometry Methods for Intact Protein Analysis and Peptide Mapping of Adeno-Associated Virus Proteins
Source: Hum Gene Ther. 2021 Dec 16;32(23-24):1501–11. doi: 10.1089/hum.2021.046 (PMC8742267; doi:10.1089/hum.2021.046)
Supplement: Supplemental data [file Suppl_TableS3.pdf]

Peak	Identity	1A	1B	2A
**1**	VP2	65,282	65,283	65,282
	VP2+1P	65,364	65,364	65,359
	VP2+2P	65,454	65,449	65,460
**2**	VP3’	59,462	59,462	59,462
**3**	VP3	59,462	59,462	59,462
**4**	VP3 clip	56,124	56,124	56,124
**5**	VP1	80,337	80,337	80,337
	VP1+1P	80,422	80,419	80,418
**6**	VP1’+1P	80,425	80,420	80,423
	VP1’+2P	80,514	80,505	80,499

Table S3. Observed masses and putative assignment of the separated VPs and their phosphorylated forms in the RPLC-MS analysis of AAV5. Larger mass errors were observed in the masses of the phosphorylated forms due to the lower abundances. The other co-eluted modifications such as deamidation and oxidation might also contribute to the mass error.
