# Supplementary material for: Optimized Reversed-Phase Liquid Chromatography/Mass Spectrometry Methods for Intact Protein Analysis and Peptide Mapping of Adeno-Associated Virus Proteins
Source: Hum Gene Ther. 2021 Dec 16;32(23-24):1501–11. doi: 10.1089/hum.2021.046 (PMC8742267; doi:10.1089/hum.2021.046)
Supplement: Supplemental data [file Suppl_FigureS3.pdf]

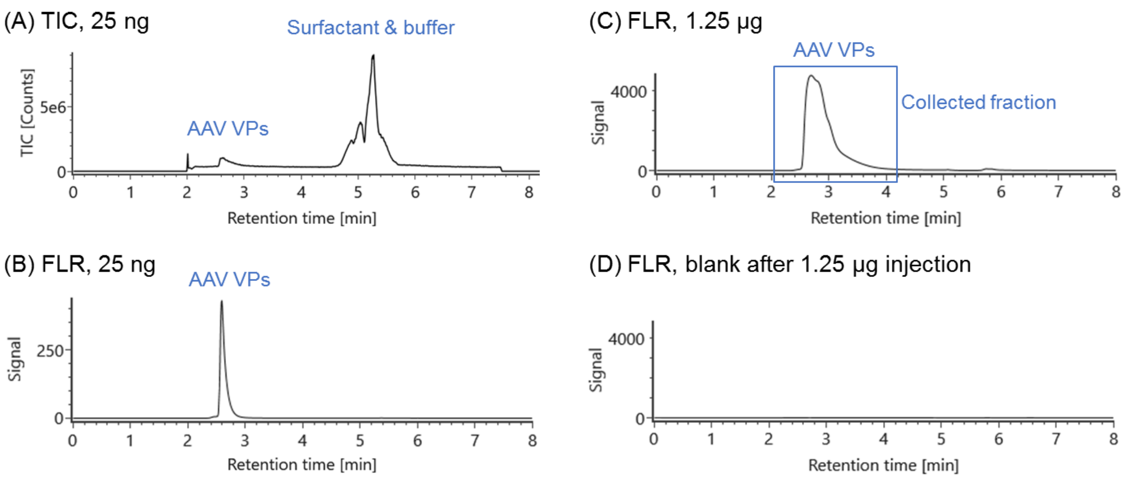

Figure S3. The removal of surfactant using denatured SEC. Using the developed 8-min method, AAV VPs were separated from the surfactant and other excipient as shown in (A) The TIC of 25 ng AAVs. (B) Under FLR detection, only the peak at 2.68 min were observed, confirming the peaks eluted after 4 min in (A) did not contain proteins. (C) The eluent of 1.25 μg AAVs was collected in the range of the blue rectangle and used in the following enzymatic digestion, while minimal carryover was observed in (D) a blank injection after fraction collection. The protein recovery was calculated to be 98.6% based on the area of the collected fraction over all peaks observed in (C) and (D). Injection volume was 25 μL which can be adjusted based on the concentration of AAV samples.
