# Supplementary material for: Optimized Reversed-Phase Liquid Chromatography/Mass Spectrometry Methods for Intact Protein Analysis and Peptide Mapping of Adeno-Associated Virus Proteins
Source: Hum Gene Ther. 2021 Dec 16;32(23-24):1501–11. doi: 10.1089/hum.2021.046 (PMC8742267; doi:10.1089/hum.2021.046)
Supplement: Supplemental data [file Suppl_FigureS4.pdf]

1: 1 to 70	SFVDHPPDWL	EEVGEGLREF	LGLEAGPPKP	KPNQQHQDQA	RGLVLPGYNY	LGPGNGLDRG	EPVNRADEVA
1: 71 to 140	REHDISYNEQ	LEAGDNPYLK	YNHADAEFQE	KLADDTSFGG	NLGKAVFQAK	KRVLEPFGLV	EEGAKTAPTG
1: 141 to 210	KRIDDHFPKR	KKARTEEDSK	PSTSSDAEAG	PSGSQQLQIP	AQPASSLGAD	TMSAGGGGPL	GDNNQGADGV
1: 211 to 280	GNASGDWHCD	STWMGDRVVT	KSTRTWVLPS	YNNHQYREIK	SGSVDGSNAN	AYFGYSTPWG	YFDFNRFHSH
1: 281 to 350	WSPRDWQRLI	NNYWGFRPRS	LRVKIFNIQV	KEVTVQDSTT	TIANNLTSTV	QVFTDDDYQL	PYVVGNGTEG
1: 351 to 420	CLPAFPPQVF	TLPQYGYATL	NRDNTENPTE	RSSFFCLEYF	PSKMLRTGNN	FEFTYNFEEV	PFHSSFAPSQ
1: 421 to 490	NLFKLANPLV	DQYLYRFVST	NNTGGVQFNK	NLAGRYANTY	KNWFPGPMGR	TQGWNLGSGV	NRASVSAFAT
1: 491 to 560	TNRMELEGAS	YQVPPQPNGM	TNNLQGSNTY	ALENTMIFNS	QPANPGTTAT	YLEGNMLITS	ESETQPVNRV
1: 561 to 630	AYNVGGQMAT	NNQSSTTAPA	TGTYNLQEIV	PGSVWMERDV	YLQGPIWAKI	PETGAHFHPS	PAMGGFGLKH
1: 631 to 700	PPPMMLIKNT	PVPGNITSFS	DVPVSSFITQ	YSTGQVTVEM	EWELKKENSK	RWNPEIQYTN	NYNDPQFVDF
1: 701 to 723	APDSTGEYRT	TRPIGTRYLT	RPL				

Figure S4. Observed PTM sites using the developed peptide mapping method, including acetylation (highlighted in orange), deamidation (highlighted in red), oxidation (highlighted in green), methylation (highlighted in blue), and phosphorylation (highlighted in magenta). Both phosphorylation and acetylation were observed on the N-terminal serine of VP1.
